# Falso hipertiroidismo por interferencia en inmunoanálisis

**DOI:** 10.1515/almed-2020-0047

**Published:** 2020-09-02

**Authors:** Clara Jiménez García, Piedad Ortega Fernández, María Eugenia Torregrosa Quesada, Victoria González Bueno, María Teresa Botella Belda, Rocío Alfayate Guerra

**Affiliations:** Laboratorio de Análisis Clínicos, Hospital General Universitario de Alicante, Alicante, España; Laboratorio de Análisis Clínicos, Hospital General Universitario de Elche, Elche, España; Unidad de Hormonas, Hospital General Universitario de Alicante, Alicante, España

**Keywords:** anticuerpos heterófilos, hipertiroidismo, interferencia de inmunoensayo

## Abstract

**Objetivos:**

Los inmunoensayos utilizados para evaluar la función tiroidea son vulnerables a diferentes tipos de interferencias que pueden afectar a las decisiones clínicas.

**Caso clínico:**

Presentamos el caso de una mujer de 37 años que acudió a nuestro hospital para su revisión médica anual por presentar hipotiroidismo iatrogénico tras tratamiento con radioiodo. La paciente se encontraba asintomática, sin signos sugestivos de enfermedad tiroidea. Sin embargo, en el estudio analítico destacó el resultado de su perfil tiroideo: TSH 7,75 mU/L, FT4 > 7,7 ng/dL.

**Conclusiones:**

Ante resultados discordantes entre la clínica y el patrón bioquímico obtenido, se consideró la posibilidad de una interferencia metodológica. Se realizó una revisión detallada de las principales causas de interferencias y se realizaron una serie de procedimientos por parte del laboratorio para descartar la presencia de interferencias en la TSH y la FT4, comprobándose finalmente que había distintos agentes interferentes que estaban afectando a las mediciones de las hormonas tiroideas libres y a la TSH de la paciente.

## Introducción

Las pruebas para la evaluación de la función tiroidea son tirotropina (TSH), tiroxina libre (FT4) y triyodotironina libre (FT3). En determinadas situaciones la relación entre la TSH y las hormonas tiroideas no se ajusta a los mecanismos de feed-back esperados y la causa puede ser debida tanto a un proceso fisiopatológico como a interferencias metodológicas. Los inmunoensayos utilizados para evaluar la función tiroidea son vulnerables a diferentes interferencias que pueden afectar a las decisiones clínicas [1].

## Caso clínico

Mujer de 37 años, en tratamiento con levotiroxina, acudió a nuestro hospital (Laboratorio 1) para su revisión anual por presentar hipotiroidismo iatrogénico tras tratamiento con radioiodo.

La paciente estaba asintomática, sin signos sugestivos de enfermedad tiroidea. En el estudio analítico destacó: TSH 7,75 mU/L (VR 0,38–4,84), FT4 > 7,7 ng/dL (VR: 0,8–1,8) y FT3 de 7,6 pg/mL (VR: 1,8–4,6 pg/mL). Las tres pruebas fueron medidas en el autoanalizador Cobas 8000® (Roche Diagnostics®) mediante inmunoensayo electroquimioluminiscente. Se confirmó el estado eutiroideo mediante la determinación de la proteína transportadora de hormonas sexuales (SHBG), cuyo valor fue 38.7 nmol/L (VR: 11–124).

La presencia de concentraciones elevadas de hormonas tiroideas, con valor normal o elevado de TSH, en ausencia de clínica compatible con hipertiroidismo y descartada la interferencia farmacológica exige considerar la posibilidad de una interferencia metodológica. Se revisaron las principales causas de interferencias en las mediciones de TSH y FT4: macro-TSH, anticuerpos heterófilos, auto anticuerpos antitiroideos, biotina, anticuerpos anti-estreptavidina o anti-rutenio [2], [3], [4], [5], [6], [7]. Se realizó el siguiente procedimiento para descartar la presencia de interferencias [8]:

1) Se confirmó el resultado repitiendo la prueba en la muestra con el mismo método, para descartar un problema de pipeteo, lavado inadecuado, agregados o burbujas.
2) Se repitieron las mediciones con una nueva muestra, obteniéndose los mismos resultados.
3) Se repitieron las mediciones con otro método diferente. Para ello se envió la muestra a otros laboratorios que empleaban una plataforma analítica diferente. Los métodos y resultados aparecen en la Tabla 1.
4) Se hicieron diluciones seriadas para medir la TSH. Se realizaron diluciones de la muestra con el diluyente recomendado por el fabricante al 1:2, 1:4, y 1:8. No se observó pérdida de linealidad en las sucesivas diluciones.
5) Se descartó la presencia de macro-TSH mediante la precipitación con polietilenglicol (PEG 6000) al 20%, obteniéndose un porcentaje de recuperación de 114%, aceptable entre 80 y 120%.
6) Se cuantificó el factor reumatoide (FR) cuyo resultado fue <20 UI/mL (VR: <20 UI/mL).
7) Se estudió la presencia de anticuerpos heterófilos mediante la incubación de la muestra en tubos de bloqueo con HBT “Heterophilic Blocking Tube” (Scantibodies®) (Tabla 2).
8) Se realizaron los anticuerpos anti-microsomales y anticuerpos anti-receptor de TSH. Siendo ambos negativos.
9) Envío de la muestra al Departamento de Investigación y Desarrollo de Roche Diagnostics.

**Tabla 1:** Métodos y resultados obtenidos en los distintos laboratorios.

Laboratorio	Equipo (casa comercial)	TSH, mU/L	FT4, ng/dL
1	Cobas (Roche)	7,7	>7,7
2	Vitros (Ortho)	23,7	0,8
3	Arquitect (Abbott)	49,1	0,6

TSH, tirotropina; FT4, tiroxina.

**Tabla 2:** Resultados obtenidos antes y después de procesar las muestras en tubos HBT.

	Laboratorio 1 (Roche-Cobas)	Laboratorio 2 (Ortho-Vitros)	Laboratorio 3 (Abbott-Arquitect)
TSH, mU/L	7,7	23,7	49,1
FT4, ng/dL	>7,7	0,8	0,6
Scantibodies			
TSH, mU/L	7,5	20,7	55,4
FT4, ng/dL	1,0	0,7	0,7

TSH, tirotropina; FT4, tiroxina; HBT, Heterophilic Blocking Tube.

## Discusión

Los inmunoensayos son los métodos más utilizados para valorar la función tiroidea, sin embargo, no están exentos de interferencias que dan lugar a resultados erróneos, tanto sobrestimando como subestimando los valores reales. No detectadas a tiempo pueden llevar a un diagnóstico incorrecto y a un tratamiento inadecuado, con las consecuentes implicaciones clínicas derivadas de dichos resultados [2], [3].

En nuestro caso, y una vez descartadas las posibles causas preanalíticas y fisiopatológicas, se consideró que los resultados discordantes de TSH y FT4 podían ser debidos a una interferencia método-dependiente.

La repetición de las determinaciones en plataformas analíticas que utilizan un inmunoensayo quimioluminiscente (Ortho-Vitros y Abbott-Arquitect) con diferentes anticuerpos confirmaron la sospecha de la existencia de una interferencia analítica, ya que se observaron diferentes resultados según el método empleado. En nuestro laboratorio, la TSH se midió mediante un método que utiliza anticuerpos quiméricos con menos posibilidad de interferencia. El resultado fue distinto por todos los métodos lo que sugiere una posible interferencia que podría afectar a todos o a dos de ellos.

La medición de FT4 fue realizada mediante método competitivo, siendo de un paso en los laboratorios 1 y 2, y de dos pasos en el laboratorio 3. Los métodos en dos pasos están sujetos a menos interferencias. La repetición de los análisis en un sistema que utilice una metodología diferente es una herramienta útil en la valoración de hormonas tiroideas discordantes. El resultado de FT4 en los laboratorio 2 y 3 confirmó que el resultado de FT4 de nuestro método estaba sujeto a interferencia.

Es conocida la falta de la linealidad ante la presencia de un agente interferente. Su utilidad es limitada ya que un 40% de las muestras con anticuerpos endógenos conocidos pueden mostrar linealidad [9], además no es aplicable a todas las magnitudes; por lo que este test no debe ser utilizado como única prueba para comprobar el resultado de un inmunoensayo o excluir la presencia de anticuerpos endógenos interferentes. En nuestro caso, no se apreció una pérdida de linealidad en las sucesivas diluciones de la TSH. Este test no se puede usar para FT4 ya que los análisis para las hormonas libres no permiten la dilución porque se altera el equilibrio entre ellas y las proteínas de transporte.

La macro TSH está formada por la unión de TSH a una inmunoglobulina formando un inmunocomplejo que puede interferir en la medición de ésta. Su presencia da lugar a resultados falsamente elevados, dependiendo de los anticuerpos utilizados en el inmunoanálisis. El tratamiento con PEG permite precipitar los complejos de elevado peso molecular. Se realizó la precipitación de la muestra con PEG en todos los laboratorios, descartándose la presencia de macro-TSH al obtener un porcentaje de recuperación similar en todos ellos.

El FR abarca un grupo heterogéneo de autoanticuerpos dirigidos contra los determinantes antigénicos de la región Fc de las moléculas de IgG. Los pacientes con artritis reumatoide u otras enfermedades autoinmunes pueden presentar concentraciones elevadas de FR, que puede unirse a los anticuerpos del reactivo dando lugar a resultados falsamente elevados [10]. En nuestro caso, se descartó la interferencia al ser indetectable.

Los anticuerpos heterófilos son anticuerpos humanos dirigidos contra inmunoglobulinas de otras especies animales que pueden producir interferencias método-específicas. Su prevalencia ha aumentado debido al empleo de fármacos biológicos con anticuerpos monoclonales para uso diagnóstico o de tratamiento [11]. Se estima que existe una incidencia de interferencia por anticuerpos heterófilos entre el 0.4 y 4% [12], [13] a pesar de que las casas comerciales adicionan a los reactivos del ensayo agentes bloqueadores a base de anticuerpos para reducir dicha interferencia. Ya que la eliminación completa de la misma no siempre es posible se pueden utilizar los sistemas de bloqueo de anticuerpos heterófilos que contienen aglutinantes específicos que los inactivan. No obstante, hay que tener en cuenta que estos sistemas de bloqueo son dependientes tanto del método como de la técnica. En nuestro caso, se estudió la presencia de anticuerpos heterófilos utilizando el sistema HBT Scantibodies, idóneos para nuestra plataforma. Tras procesar la muestra con el agente bloqueante se confirmó la interferencia por anticuerpos heterófilos en el ensayo de FT4 en el laboratorio 1, ya que los resultados se normalizaron tras el bloqueo siendo similares a los obtenidos en las otras dos plataformas analíticas.

La biotina a dosis altas (>5 mg/día), puede interferir en los inmunoanálisis que utilizan el sistema biotina-estreptavidina en el diseño del ensayo para amplificar la señal de detección, tanto en los métodos competitivos (FT4) como en los no competitivos (TSH), pudiendo originar resultados falsamente elevados o disminuidos respectivamente [14]. En Roche las determinaciones de TSH, FT4 y FT3 pueden ser afectadas por el exceso de biotina. En Ortho solamente la TSH se ve afectada ya que las técnicas para FT4 y FT3 no utilizan biotina. Los inmunoensayos de Abbott al no utilizar los sistemas biotina-estreptavidina no se ven afectados pudiendo ser un método de elección para identificar indirectamente la interferencia por biotina [2]. Nuestra paciente negaba la toma de cualquier suplemento con biotina.

Los anticuerpos anti-rutenio se producen cuando el rutenio medioambiental está en concentraciones elevadas [15]. Roche introdujo en 2006 cambios en los reactivos que no llegaron a eliminar la interferencia pero este año se han reformulado los reactivos de TSH, FT4 y FT3 consiguiendo eliminarla.

Para completar el estudio y realizar una evaluación más detallada, la muestra fue remitida al Departamento de Investigación y Desarrollo de Roche Diagnostics. Los resultados obtenidos no informaron de interferencia por biotina ni por anticuerpos anti-rutenio, pero sí confirmaron la presencia de una interferencia sin concluir de que tipo.

Entre las limitaciones del estudio podemos destacar la imposibilidad para explicar los diferentes resultados de TSH obtenidos en los distintos laboratorios, si bien es cierto que en ocasiones no es posible identificar la interferencia que produce los resultados discordantes.

Estamos ante un caso complejo que incluye distintos interferentes que afectan a la medición de hormonas tiroideas libres y TSH.

## Conclusiones

Ante resultados discordantes se debe considerar la presencia de interferencias analíticas asociadas a los inmunoensayos automatizados, por lo que hay que conocer bien la plataforma utilizada para orientar su etiología, pero a veces no se llega a conocer la verdadera naturaleza del agente interferente.

Es responsabilidad del laboratorio clínico identificar y notificar la presencia de interferencias, para minimizar el impacto que pudieran tener en las decisiones clínicas. La comunicación continua entre especialistas del laboratorio, clínicos y fabricantes es esencial para identificar y prevenir estas situaciones.
